# Ferroelectric Devices for In‐Memory and In‐Sensor Computing

**DOI:** 10.1002/advs.202600007

**Published:** 2026-04-23

**Authors:** Hong Fang, Ronghuan Xie, Qiang Cao, Shishen Yan, Xiaolin Wang, Limei Zheng

**Affiliations:** ^1^ Spintronics Institute University of Jinan Jinan P. R. China; ^2^ School of Physics Shandong University Jinan P. R. China; ^3^ Institute For Superconducting and Electronic Materials Australian Institute for Innovative Materials ARC Centre of Excellence in Future Low‐Energy Electronics Technologies University of Wollongong Wollongong New South Wales Australia

**Keywords:** artificial synapse, ferroelectric devices, in‐memory computing, in‐sensor computing, neuromorphic sensors

## Abstract

Inspired by biological neural and sensory systems, the in‐memory computing and in‐sensor computing paradigms have emerged, which integrate computation with memory and processing with sensor respectively, offering a promising solution to address latency and power bottlenecks of traditional von Neumann architectures. Neuromorphic devices such as artificial synapse and neuromorphic sensors are the core components of these innovative computing paradigms. Among various types of neuromorphic devices, ferroelectric devices can not only emulate synaptic weight updates via electric‐field‐induced dynamic modulation of conductance, but also sense and process multimodal physical stimuli, such as light, mechanical force, and heat. Such multifunctionality enables the integration of sensing, memory, and computation at the device level, positioning ferroelectric devices as an ideal platform for neuromorphic computing and sensing systems. Herein, we present a systematic review of ferroelectric neuromorphic devices for in‐memory computing and in‐sensor computing. The applications of ferroelectric devices as artificial synapses in in‐memory computing are summarized. Furthermore, we examine the applications of ferroelectric devices as sensing elements in in‐sensor computing systems and summarize the latest research advances in these fields. Finally, this review outlines the key challenges faced by ferroelectric neuromorphic devices and proposes future development directions to promote their practical applications.

## Graphical abstract

Inspired by biological systems, in‐memory and in‐sensor computing overcome von Neumann bottlenecks. Ferroelectric devices can mimic synaptic functions and sense stimuli like light or force, therefore are ideal for these paradigms. This review introduces the ferroelectric devices applied for in‐memory and in‐sensor computing, covering their structures, operating mechanisms, and specific task processing.

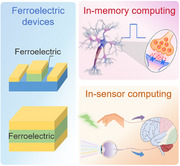

## 1 Introduction

With the advent of big data era, the rapid expansion of data‐intensive applications, such as artificial intelligence (AI) and the Internet of Things, has posed unprecedented challenges to traditional computing paradigms based on the von Neumann architectures [1, 2]. The physical separation between memory and processing units in this architecture necessitates frequent data transfer, resulting in high latency and energy consumption, which collectively form a performance bottleneck known as the “memory wall” [3]. Similar architectural bottlenecks also restrict the traditional sensor systems, where the sensing, storage, and information processing modules operate in isolation [4]. The data must migrate across multiple discrete modules, leading to redundant transmissions, excessive power consumption as well as significant latency [5]. To overcome these systemic challenges, a significant change in architectural paradigm is imperative.

With this purpose, researchers have turned to biological systems for inspiration, which inherently integrate the perception, memory, and computation. Specifically, drawing from biological neural systems, where memory and computation are intrinsically integrated and intertwined [6], the researchers have pursued brain‐inspired neuromorphic computing systems that integrate computing functions into memory units, a paradigm known as “in‐memory computing” [7, 8, 9]. Furthermore, inspired by biological sensory systems, the concept of “in‐sensor computing” has emerged [10, 11]. This novel neuromorphic sensor system that integrates sensing, storing, and processing functions, aiming to achieve low‐latency and energy efficient [12, 13].

The hardware implementation of both in‐memory and in‐sensor computing relies on artificial neuromorphic devices capable of emulating biological functions [14, 15, 16, 17]. Artificial synapses and sensory devices serve as the fundamental building blocks for in‐memory and in‐sensor computing, respectively [18]. In the in‐memory computing systems, artificial synapses store and update synaptic weights, enabling learning and adaptive functionalities [19, 20, 21]. In the in‐sensor computing systems, the sensory devices not only convert physical stimuli into electrical signals, but also execute fundamental information‐processing tasks, such as feature extraction, data compression, etc. [22, 23, 24]. To achieve these functions and meet the growing demands of machine learning algorithms and emerging intelligent systems, it is crucial to continuously improve and explore novel neuromorphic devices [25, 26].

Among the intensively studied neuromorphic devices, ferroelectric devices stand out for their distinctive advantages such as excellent controllability, ultrafast read/write speeds (∼ns) [27, 28], ultrawide dynamic conductance range (>10^7^) [29, 30], ultralow power consumption (∼fJ) [31, 32, 33], and outstanding endurance (∼10^9^ cycles) [34, 35], etc. A pioneering work reported by Chanthbouala et al. in 2012 demonstrated the first ferroelectric memristor‐a ferroelectric tunnel junction (FTJ) [36]. The multi‐level and continuously tunable conductance states can be realized by controlling partial polarization switching in this FTJ, thereby successfully simulating the long‐term potentiation (LTP) and long‐term depression (LTD) of synaptic weight updates. Since then, various ferroelectric device architectures, such as ferroelectric field‐effect transistors (FeFETs) and ferroelectric diodes (FDs), have been extensively explored for neuromorphic applications [37, 38]. Furthermore, by achieving dynamic modulation of device conductance, researchers have simulated more diverse forms of synaptic plasticity, such as spike‐timing‐dependent plasticity (STDP), short‐term synaptic plasticity (STSP), and adaptive plasticity, thereby advancing the development of neuromorphic computing [36, 39]. Additionally, the polarization state can interact with external physical stimuli (e.g., light, mechanical force, heat) through the coupling effects such as photovoltaic, piezoelectric, pyroelectric, etc. This interaction produces distinct electrical signals that facilitate in‐sensor signal perception and processing [40, 41, 42, 43].

For ferroelectric materials, early synaptic devices predominantly employed traditional perovskite ferroelectrics, which often faced challenges such as insufficient compatibility with CMOS processes and limited scalability [44]. In recent years, the emergence of new ferroelectric systems, such as HfO_2_‐based ferroelectrics and two‐dimensional ferroelectric materials, has provided new pathways to overcome these bottlenecks [45, 46]. Concurrently, advances in material systems have enabled more diverse device architectures, such as multimodal devices capable of responding to multiple external stimuli [47].

In a word, such intrinsic integration of sensing, memory, and computation within a single device positions ferroelectric neuromorphic devices as attractive building blocks for both in‐memory and in‐sensor computing paradigms. This review focuses on the ferroelectric neuromorphic devices and their applications in in‐memory and in‐sensor computing paradigms. First, we introduce ferroelectric artificial synapses for in‐memory computing, emphasizing device structures, operating mechanisms, synaptic functions as well as their applications in spatial, temporal, and spatiotemporal learning scenarios. Then, we explore ferroelectric sensory devices for in‐sensor computing, analyzing the unique advantages of ferroelectric devices and recent progress in perception–memory–computation integration. Finally, the challenges and prospects for the further development of ferroelectric neuromorphic devices are proposed.

## 2 Ferroelectric Devices for In‐Memory Computing

In‐memory computing integrates computing functions into storage units to eliminate the need for frequent data transfer between storage and computing units, thereby significantly reducing energy consumption and improving computing efficiency [48]. Such a bio‐inspired architecture is primarily composed of artificial synapses and neurons. Among these, artificial synapses are responsible for signal transmission while also integrating memory and processing capabilities, making them the core components of the system.

In recent years, researchers have developed various neuromorphic devices to emulate synaptic functions, including resistive random access memory (RRAM), phase change memory (PCM), magnetic random access memory (MRAM), and ferroelectric memristive devices [49]. Among these, RRAM operates mainly through the formation and rupture of conductive filaments to achieve non‐volatile resistive switching. However, the inherent stochasticity in filament formation leads to considerable device‐to‐device and cycle‐to‐cycle variability, which remains a major challenge for practical applications [50]. The PCM relies on reversible phase transitions between amorphous and crystalline states to modulate resistance, yet its thermally driven switching mechanism results in inherent unipolar operation and high‐power consumption due to Joule heating [51]. The MRAM functions by altering the spin orientation in magnetic materials, a process that requires high driving currents during write operations and consequently entails significant energy expenditure [52]. In contrast, ferroelectric neuromorphic devices utilize the switching of ferroelectric polarization under an electric field to simulate synaptic weight updates and information processing. Due to the inherent advantages of polarization reversal, they exhibit excellent controllability, bipolar resistive switching, a large conductance change range, and ultra‐low energy consumption, positioning them as one of the most promising candidates for in‐memory computing.

In this section, we will briefly introduce the application of ferroelectric neuromorphic devices as synaptic elements in in‐memory computing. The discussion covers their typical structure, operating mechanisms, and the implementation of basic synaptic behaviors, along with demonstrations in different computing scenarios.

### 2.1 Ferroelectric Artificial Synapses: Structure and Working Mechanism

In ferroelectric neuromorphic devices, the conductance can be reversibly and continuously modulated by an external electric field. This continuous variation in conductance can be utilized to simulate the update rules of synaptic weights, thereby enabling the successful accomplishment of computational or learning tasks.

#### 2.1.1 Fundamental Synaptic Behaviors of a Biological Synapse

**FIGURE 1:**
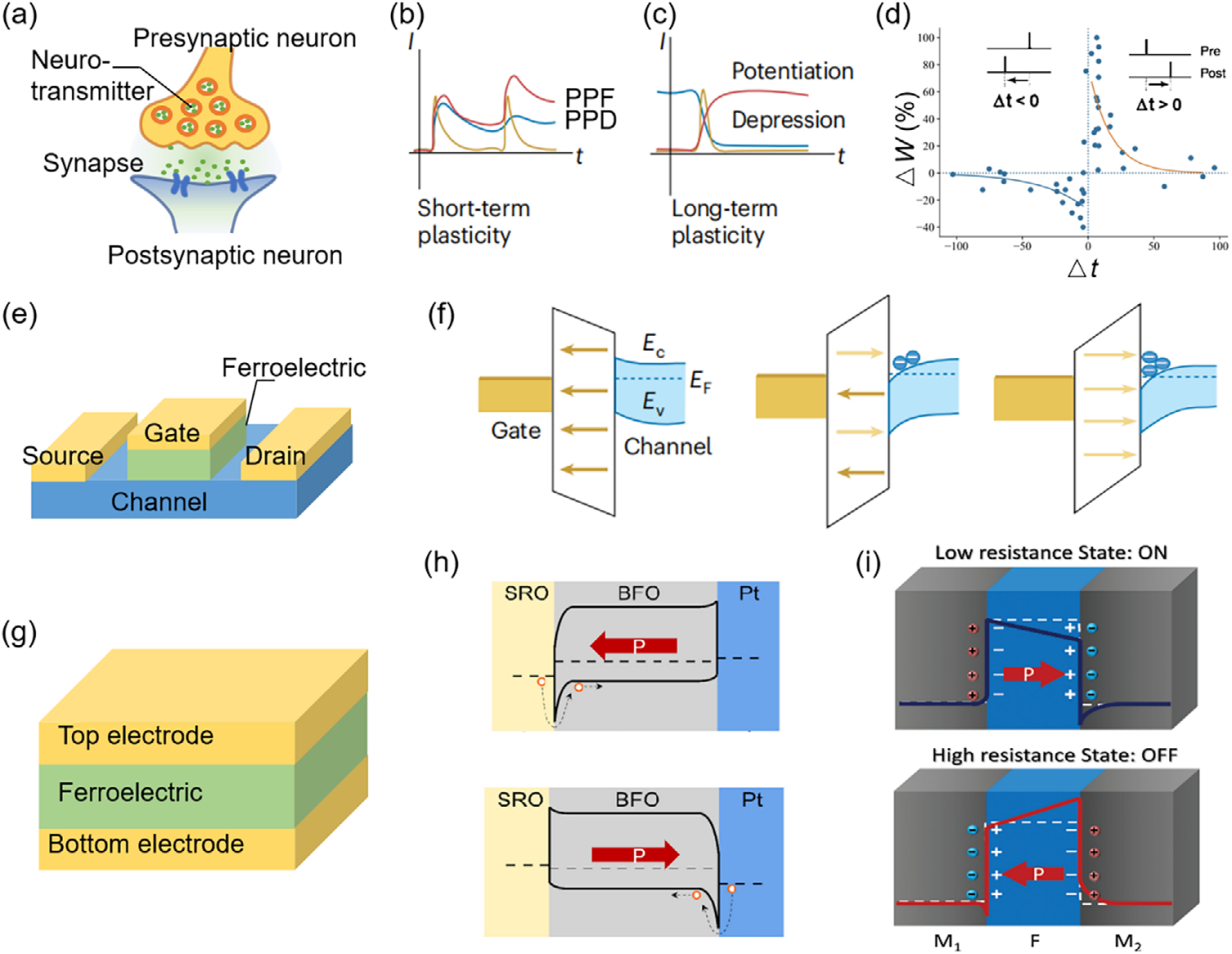
The structure and working mechanism of ferroelectric artificial synapses. (a) Schematic diagram of a biological synapse. (b–d) The synaptic plasticity, including PPF/PPD, LTP/LTD, and STDP. Reproduced with permission [56]. Copyright 2025 Spring Nature. (e) Structure diagram and (f) schematic diagram of the conductance modulation of the FeFET. Reproduced with permission [56]. Copyright 2025 Spring Nature. (g) Structure diagram of the two‐terminal device. (h,i) The resistive switching mechanism in FD and FTJ, respectively. Reproduced with permission [64, 65]. Copyright 2005 American Physical Society. Copyright 2023 Spring Nature (CC BY).

In biological nervous systems, synapse refers to the element through which the impulse of one neuron is transmitted to another, playing a key role in information transmission [53]. Figure 1a illustrates the typical structure of a biological synapse. When an action potential propagates to the presynaptic terminal, neurotransmitters are released into the synaptic cleft and bind to receptors on the postsynaptic membrane, generating the excitatory or inhibitory currents (EPSCs or IPSCs) [54]. Notably, the postsynaptic current that represents the synaptic weight is not static but can be dynamically adjusted based on relative neural activity, a phenomenon known as synaptic plasticity. According to the synaptic weight retention time, the synaptic plasticity is generally categorized into long‐term synaptic plasticity (LTSP) and STSP.

STSP typically lasts from hundreds to thousands of milliseconds, leading to transient changes in synaptic efficacy. Without sustained presynaptic stimulation, synaptic strength decays back to baseline. STSP includes short‐term potentiation and depression, both contribute to short‐term memory processes in the brain, such as filtering irrelevant information [55]. Paired‐pulse facilitation (PPF) and paired‐pulse inhibition (PPD) are two representative forms of STSP, which are crucial for processing temporal information. Specifically, as depicted in Figure 1b [56], PPF refers to the phenomenon where, when two consecutive pulse stimuli are applied within a short time period, the amplitude of the EPSC induced by the second pulse is significantly higher than that induced by the first pulse. In contrast, PPD is characterized by a lower amplitude of the IPSC induced by the second pulse compared to that induced by the first pulse.

Conversely, LTSP refers to persistent changes in synaptic weight induced by specific neural activity (Figure 1c). LTSP includes LTP and LTD, being crucial for learning and memory in the brain [57]. STDP is a type of LTSP associated with the relative timing between presynaptic and postsynaptic spikes [58, 59]. As depicted in the Figure 1d, the synaptic weight exhibited an exponential variation with the Δ*t* (where Δ*t* is the relative time interval of the pre‐ and postsynaptic spikes). Specifically, when a presynaptic spike is triggered momentarily ahead of a postsynaptic spike (Δ*t*>0), the synaptic weight increased and LTP occurred [60]. In contrast, when Δ*t*<0, the synaptic weight decreased, resulting in LTD.

The coexistence of basic synaptic plasticity across multiple time scales endows the biological neural system with remarkable adaptability [61]. Furthermore, synaptic plasticity exhibits historical dependence, where historical stimulus patterns can modulate the subsequent plasticity [62]. Such adaptive synaptic plasticity plays a crucial role in the remodeling process of the human sensory cortex and can be further used to perform spatiotemporal learning tasks [63].

#### 2.1.2 Typical Structures of Ferroelectric Artificial Synapses

Based on the device structure, the ferroelectric artificial synapses can be categorized into three main types: FeFETs, FDs, and FTJs.

As shown in Figure 1e, the FeFETs are representative three‐terminal devices whose structure typically comprises a semiconductor channel layer, a ferroelectric layer, and source, drain, and gate electrodes [66]. When used as an artificial synapse, the gate electrode acts as the presynaptic terminal, receiving input voltage pulses to changing ferroelectric domain states, through which the channel conductance can be modulated to simulate the updating of synaptic weights. A schematic diagram of the conductance modulation of the FeFET is presented in Figure 1f [56]. Taking an *n*‐type channel as an example: when the ferroelectric polarization direction is upward (toward the gate electrode), polarization‐induced negative bound charges accumulate at the interface, forming a carrier depletion layer in the channel and leaving the device in a high‐resistance state. Conversely, with downward polarization, carriers accumulate in the channel, and the device switches accordingly from a high‐resistance to a low‐resistance state. Thus, by applying a specific sequence of pulsed voltages, the gradual switching of ferroelectric domains can be driven, achieving continuous modulation of carrier distribution and device conductance [67]. This, in turn, allows the FeFET to function as an artificial synapse.

In contrast, both FDs and FTJs are two‐terminal devices, generally consisting of two electrodes separated by a ferroelectric barrier (Figure 1g). With their top and bottom electrodes corresponding to the presynaptic and postsynaptic membranes, respectively, dynamic updates of synaptic weights are achieved via changes in device conductance. For FDs, the ferroelectric layer is relatively thick, the top and bottom electrodes exhibit an asymmetric screening effect on the polarization charges at the interface, inducing variations in the interfacial barrier [68, 69]. The dynamic modulation of interfacial barrier height through polarization direction can significantly affect the injection efficiency of electrons or holes (Figure 1h), thereby enabling the modulation of device conductance [65, 70]. In contrast, FTJs feature an ultrathin ferroelectric layer less than 5 nm, where electron transport is dominated by quantum tunneling as the primary mechanism [71]. In FTJs, the average barrier height is a key factor governing electron tunneling probability, with a reduced average height leading to a significant increase in tunneling probability (Figure 1i) [64]. Notably, when a semiconductor electrode is employed on one side of the FTJ, the formation of a depletion layer at the semiconductor‐ferroelectric interface enables further modulation of the tunneling barrier width [72, 73]. Through the synergistic modulation of barrier height and width, FTJs can achieve continuous tuning of device conductance, providing an effective approach for simulating synaptic weight updates.

Above three types of ferroelectric artificial synapses, due to fundamental differences in their operating principles and device structures, exhibit significant divergence in performance and application. The FeFET is distinguished by the physical separation between its read terminals (source/drain) and write terminal (gate). Compared with the two‐terminal devices, this design offers several advantages: it enables parallel signal processing and synaptic weight updating; it significantly reduces the leakage current and lowers the device's power consumption; its structure is highly similar to conventional silicon‐based FETs, granting it excellent CMOS compatibility. Moreover, in multi‐gate FeFETs, each input terminal can independently receive signals and collectively modulate the channel conductance, providing a physical basis for emulating complex synaptic functions like hetero‐synaptic plasticity [74, 75]. However, during conductance modulation, the high electric field concentrated at the ferroelectric/channel interface raises serious endurance concerns [76]. The separated terminals also result in a more complex device structure and fabrication process.

In comparison, the two‐terminal devices including the FDs and FTJs offer the advantage of structural simplicity. For the FDs, the relatively thick ferroelectric layer leads to a smaller internal depolarization field, which contributes to superior non‐volatility [40, 77]. Moreover, under specific bias conditions, FDs exhibit selector‐like rectification due to asymmetric modulation of the interface barrier, which is advantageous for high‐density crossbar integration. Nevertheless, polarization‐induced modulation of the interface barrier in these devices remains limited, making it difficult to achieve a wide dynamic range of conductance. In contrast, the FTJs are capable of achieving multiple distinguishable conductance states over a wide dynamic range by synergistically modulating the height and width of the tunneling barrier [78]. Furthermore, the progressive switching of ferroelectric domains enables continuous and linear tunability of the conductance states, providing significant advantages for realizing high‐precision neuromorphic computing. However, the reliable fabrication of large‐area, uniform ultrathin ferroelectric films remains a key challenge, keeping FTJs at the forefront of current research.

In summary, the characteristics of these three types of ferroelectric synaptic devices are complementary and mutually reinforcing. The FTJs, with their excellent linearity and controllability, are well suited for building high‐precision analog synaptic arrays for core computing tasks. The FDs can be employed to implement non‐volatile storage and selection functions in hardware neural networks. Meanwhile, the FeFETs, benefiting from its multi‐terminal design and strong compatibility with CMOS processes, are better suited for integration into peripheral circuits to emulate complex synaptic functions.

#### 2.1.3 Typical Ferroelectric Materials in Ferroelectric Artificial Synapses

In ferroelectric synaptic devices, the functions are primarily realized through the polarization switching. Serving as the core functional layer, the properties of the ferroelectric material fundamentally determine the overall device performance. To date, a variety of ferroelectric materials have been broadly explored for neuromorphic applications, including perovskite ferroelectrics (e.g. BaTiO_3_, Pb(Zr_1‐_ *_x_* Ti*_x_*)O_3_), flexible organic ferroelectric polymers (e.g. P(VDF‐TrFE)), HfO_2_‐based ferroelectrics with excellent CMOS compatibility, and the recently‐emerged two‐dimensional (2D) ferroelectric materials (e.g. *α*‐In_2_Se_3_, CuInP_2_S_6_).

Among all the types, the perovskite ferroelectric materials are the most thoroughly studied. They adopt the typical ABO_3_ structure, whose ferroelectricity originates from structural distortions that break the centrosymmetry of the crystal lattice. Benefiting from mature fabrication processes, these materials are wellsuited for proofofconcept demonstrations and prototype research in neuromorphic systems. However, the integration with standard CMOS technology remains challenging due to the high fabrication temperature and the difficulty in achieving nanoscale miniaturization [79]. To address this limitation, HfO_2_‑based ferroelectric materials with a fluorite structure have been developed, which not only retain ferroelectricity at the nanoscale but also exhibit improved compatibility with CMOS technology. Nevertheless, their ferroelectricity relies on a metastable orthorhombic phase (Ophase), the stability of which is highly sensitive to preparation conditions, such as doping, stress, and temperature [80]. Additionally, both perovskite and HfO_2_based materials generally lack mechanical flexibility. In contrast, the ferroelectricity in organic ferroelectric polymers arises from the *β*‑phase chain conformation. These materials exhibit outstanding flexibility and biocompatibility, making them ideal for wearable systems. A major drawback is their typically high coercive field, which necessitates high operating voltages and leads to increased power consumption. Recently, 2D ferroelectric materials have emerged. Their ferroelectricity stems from in‐plane atomic displacement or symmetry breaking within interlayer stacking, offering the notable advantages of low switching voltages and minimal power consumption. With atomic‐scale thickness, dangling‐bond‐free surfaces, and van der Waals stackability, they are ideal platforms for constructing heterostructures that enable multifunctional coupling, such as ferroelectric‐photoelectric interactions [81]. However, the preparation of 2D ferroelectric devices currently relies largely on mechanical exfoliation, which impedes the scalable fabrication of large‐area arrays. Moreover, their performance is often susceptible to environmental fluctuations. The important parameters comparison of these ferroelectric materials, including their crystal structures, respective advantages, limitations, and applications in neuromorphic systems, is systematically summarized in Table 1.

**TABLE 1:** Summary and comparison of different ferroelectric materials.

Material type	Representative materials	Structural features	Advantages	Limitations	Application in neuromorphic systems
Perovskite‐type ferroelectric materials	BaTiO_3_; Pb(Zr, Ti)O_3_; BiFeO_3_	Perovskite structure	Mature fabrication process; High remanent polarization	Poor CMOS compatibility; Challenging nanoscale miniaturization	Proof‐of‐concept & prototype device development
HfO_2_‐based ferroelectric materials	Si:HfO_2_; Zr:HfO_2_; Al:HfO_2_	Fluorite structure	Excellent CMOS compatibility; Nanoscale ferroelectricity	Metastable ferroelectric phase; Sensitive to fabrication conditions	High‐density memory‐computing integrated arrays
Flexible/organic ferroelectric polymers	P(VDF‐TrFE); PVDF‐based copolymer	Semi‐crystalline polymer	Low processing temperature; High flexibility & biocompatibility	High coercive field/operating voltage; High power consumption	Flexible synaptic devices & wearable neuromorphic systems
2D ferroelectric materials	α‐In_2_Se_3_; CuInP_2_S_6_	Layered van der Waals structure	Facile hetero‐integration; Stackability & multifunctional coupling	Challenging large‐scale preparation; Environmental stability issues	Multimodal neuromorphic devices & integrated sensing‐memory‐computing systems

### 2.2 Simulation of Long‐Term Synaptic Plasticity and Its Application in Spatial Information Processing

Above all, LTSP, as the foundation of brain memory and learning, serves as a core design requirement for implementing in‐memory computing [82]. Owing to the typical non‐volatility of ferroelectric polarization, the ferroelectric devices are well‐suited for simulating long‐term synaptic behaviors, making them ideal for static spatial pattern recognition and classification tasks in artificial neural networks (ANNs) and spiking neural networks (SNNs).

**FIGURE 2:**
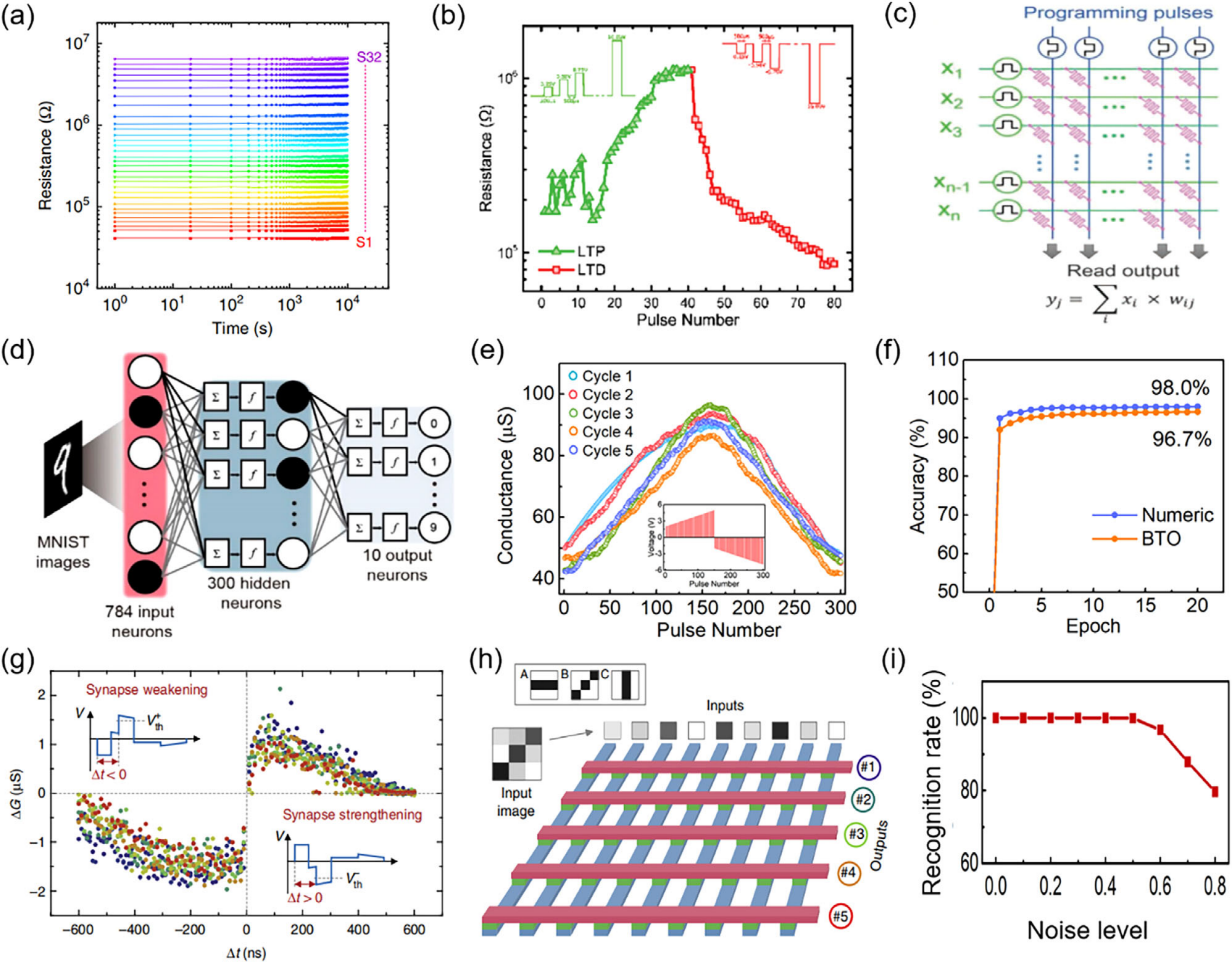
The long‐term synaptic plasticity in ferroelectric artificial synapses and its application in patterns recognition. (a) Fundamental characteristic of a ferroelectric synapse: controllable multi‐level non‐volatile conductance states. Reproduced with permission [28]. Copyright 2020 Spring Nature (CC BY) (b) LTP and LTD behaviors observed upon the application of increasing positive and negative pulse trains, respectively. Reproduced with permission [83]. Copyright 2018 John Wiley and Sons. (c) The schematic diagram of an ANN with a crossbar structure to perform the analog matrix operations. Reproduced with permission [82]. Copyright 2020 John Wiley and Sons. (d) Schematic illustration of the ANN for pattern recognition tasks. Reproduced with permission [85]. Copyright 2022 American Chemical Society. (e) Linear and symmetric LTP/LTD curves with a nonlinearity coefficient of 0.13–0.17. Reproduced with permission [78]. Copyright 2022 John Wiley and Sons. (f) The classification accuracy of FTJs‐based synapses and ideal devices. Reproduced with permission [78]. Copyright 2022 John Wiley and Sons. (g) The typical STDP curves in the ferroelectric synapses and (h) the simulated SNN. Reproduced with permission [91]. Copyright 2017 Spring Nature (CC BY). (i) The variation of the recognition rate with the noise level in the SNN based on the balanced STDP curve. Reproduced with permission [78]. Copyright 2022 John Wiley and Sons.

Figure 2a illustrates the fundamental characteristics of the ferroelectric synapses: multiple non‐volatile resistive states can be achieved in a single ferroelectric device by adjusting the upward and downward ferroelectric domains in varying proportions [28]. Moreover, by applying continuous pulse voltage with opposite polarity, LTP and LTD can be successfully simulated (Figure 2b) [83]. Furthermore, the ferroelectric synaptic devices can be arranged in a cross array to construct a complete hardware neural network (Figure 2c) [82, 84]. By utilizing the conductance value of each device to represent the synaptic weight and relying on Ohm's law and Kirchhoff's law, the vector‐matrix multiplication and multiply‐accumulate operations in ANNs can be directly performed, thereby enabling a massive parallel signal processing within the memory. Specifically, Luo et al. constructed a typical three‐layer ANN utilizing the Cross Sim platform. By using the conductance values from the LTP and LTD curves of the MoS_2_/P(VDF‐TrFE) FeFET to map synaptic weights, the neural network successfully achieved the recognition of MNIST handwritten digits with an accuracy of 86% (Figure 2d) [85].

However, the synaptic weight updates usually show poor linearity and symmetry, causing the learning accuracy of the ANN to be lower than that of software‐based computer programs [86]. This low linearity and symmetry arise from the fact that the switching process of ferroelectric domain exhibits remarkable nonlinear characteristics, specifically, the relatively slow domain nucleation stage and the rapid domain expansion stage [83, 87]. To improve its learning accuracy, a large amount of work has focused on optimizing the conductance update process to enhance their linearity and symmetry, with specific strategies including optimizing the pulse sequence parameters [33, 88, 89, 90], regulating the dynamic range of conductance variation [82], optimizing the orientation and size of ferroelectric domains [32], and introducing defects to control domain switching speed [78]. To date, the nonlinearity coefficient of conductance update in the ferroelectric artificial synapses has been as low as 0.13‐0.17 (Figure 2e) [78]. The ANN constructed based on these devices has a learning accuracy of up to 96.7%, which is already close to the ideal neural network performance of 98% (Figure 2f).

Unlike LTP and LTD, the STDP is influenced by input spike timing, making it crucial for associative learning with incomplete inputs and enabling unsupervised learning in SNNs. For instance, Bryon et al. made a significant breakthrough by successfully simulating STDP behavior in the Pt/BiFeO_3_/Ca_0.96_Ce_0.04_MnO_3_/ YAlO_3_ FTJ, as depicted in Figure 2g [91]. Furthermore, leveraging the conductance values of the STDP curves, a SNN was skillfully constructed, enabling the successful implementation of unsupervised learning for image information (Figure 2h). Subsequently, Guo and co‐workers discovered that the interface engineering can induce different STDP behaviors in FTJ‐based synaptic devices. These include STDP curves following antisymmetric Hebbian and antisymmetric anti‐Hebbian learning rules, each of which will play distinct and specific roles in different SNNs [92]. In addition, Yang et al. obtained a balanced STDP curve by regulating the domain switching behavior through oxygen vacancies [78]. When this balanced STDP curve was applied to an SNN for image recognition, robust unsupervised learning in a noisy environment can be achieved: the recognition rate still remains nearly 100% even when the noise level increased to 0.5‐times of the input amplitude (Figure 2i).

### 2.3 Simulation of Short‐Term Synaptic Plasticity and Its Application in Temporal Information Processing

These above researches have focused on the LTSP of ferroelectric synaptic devices and their application in ANNs and SNNs, successfully achieving the spatial information processing. However, in practical scenarios, the brain also needs to process a large amount of time‐related information (e.g. speech signals). To address this need for efficient temporal processing, reservoir computing (RC) has emerged as a promising approach [93, 94]. In RC systems, the reservoir serves as the core component, responsible for nonlinearly converting the input temporal signal into a high‐dimensional spatial state (reservoir state) [95]. These states depend not only on the current input but also on historical inputs, making STSP a critical requirement for hardware implementation of reservoirs [96]. In ferroelectric synaptic devices, the nonlinear dynamic responses generated by the effects such as polarization relaxation and charge trapping/detrapping at defect states enable them very suitable for simulating STSP and encoding temporal information [97, 98].

**FIGURE 3:**
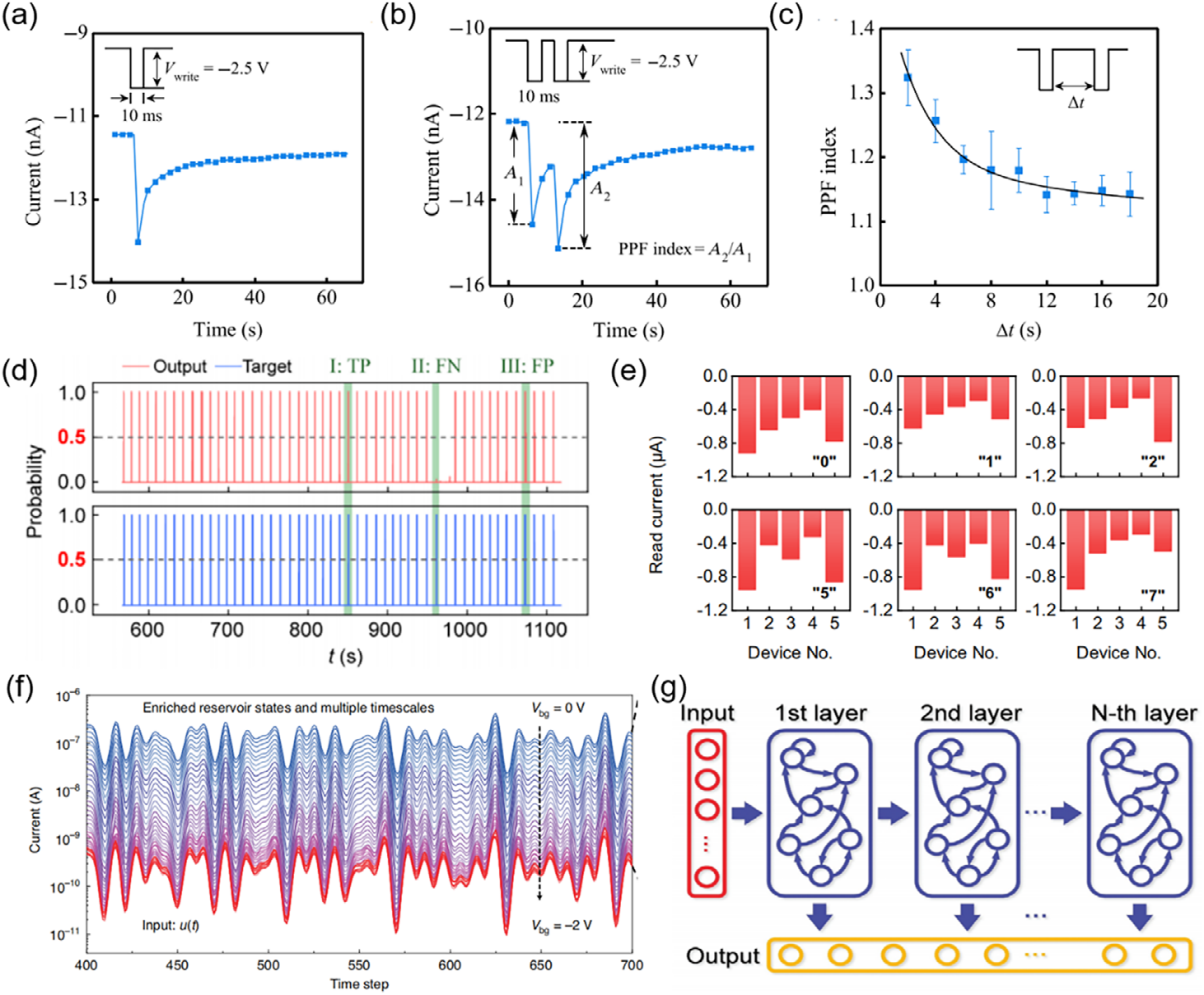
The short‐term synaptic plasticity in ferroelectric artificial synapses and its application in temporal information processing. (a) The EPSC in response to a −2.5 V (10 ms) pulse in the Pt/BiFeO_3_/SrRuO_3_ FD. Reproduced with permission [97]. Copyright 2023 American Physical Society. (b) The typical PPF behavior and (c) PPF index as a function of pulse internal (Δ*t*) in FD‐based ferroelectric synapse. Reproduced with permission [97]. Copyright 2023 American Physical Society. (d) Output signal and target of the RC system for digital voice task "2". Reproduced with permission [99]. Copyright 2024 American Chemical Society. (e) Experimentally measured reservoir states from the volatile FDs with different input images. Reproduced with permission [65]. Copyright 2023 Spring Nature (CC BY). (f) The dynamic current responses of the reservoir, where enriched reservoir states are generated with varied back‐gate voltages. Reproduced with permission [47]. Copyright 2022 Spring Nature. (g) Schematic circuit diagram and architecture of the multilayer RC system, where the output of first layer was connected to the input of second layer. Reproduced with permission [102]. Copyright 2022 John Wiley and Sons.

As illustrated in Figure 3a, when a voltage pulse (−2.5 V, 10 ms) is applied to the Pt/BiFeO_3_/SrRuO_3_ FD, a transient increase in the EPSC can be observed, which corresponds to the typical feature of biological STSP [97]. As presented in Figure 3b, the EPSC triggered by the second peak exhibited a higher response compared to that of the first one, indicating the successful emulation of PPF behavior. The PPF index is employed to characterize the influence of the first pulse stimulus on the enhancement of the second pulse. As can be observed from Figure 3c, the PPF index has an exponential relationship with the pulse interval, consistent with the biological synapses. The successful simulation of STSP demonstrates that ferroelectric devices can nonlinearly transform time‐series inputs into high‐dimensional reservoir states and can be used for efficiently processing temporal tasks in RC systems.

Currently, the ferroelectric device‐based RC systems are primarily applied to two key types of temporal information processing tasks: one involves analyzing signals with dynamic temporal characteristics (e.g., speech and images), and the other involves time‐series prediction and waveform classification tasks. For speech signal processing tasks, Yi et al. constructed a single‐node RC system using the CuInP_2_S_6_ (CIPS)/graphene heterostructure devices [99]. By dynamically regulating thermionic electron injection and transport, the typical nonlinear current response and fading memory characteristics are achieved in the ferroelectric device. Speech signals are converted into one‐dimensional voltage sequences, which are applied to the device; the resulting current output from the device forms the reservoir states, enabling efficient processing of speech signals (Figure 3d). Additionally, Chen et al. developed a representative RC system based on Pt/BiFeO_3_/SrRuO_3_ FDs, which successfully processed temporal image information [65]. In the volatile FD, through introducing an imprint field formed by oxygen vacancies to regulate the back‐switching of spontaneous polarization, the conductance decay characteristics are realized. By encoding the pixels of each row of the image as a time frame pulse sequence and applying it to the device, the resulting device current is collected to form the reservoir states representing image information. As shown in the Figure 3e, the reservoir states corresponding to different letters exhibit significant differences, validating the device's capability for temporal image processing. Moreover, to address the demand for multimodal temporal information fusion, Nie et al. achieved the bi‐directional relaxation characteristics of conductance by introducing an ion diffusion mechanism into the FTJ [100]. Building on this, they encoded image and voice multimodal signals via positive and negative polarity voltages respectively, and constructed a multimodal RC system, providing a new research approach for the collaborative analysis of cross‐modal temporal information.

Another typical category of tasks primarily focuses on time‐series prediction and waveform classification tasks. Relevant studies have further expanded the application scenarios of RC system by optimizing the dynamic characteristics and structural design of ferroelectric devices. For instance, Toprasertpong et al. reported a RC system that used the FeFET with the Hf_0.5_Zr_0.5_O_2_ ferroelectric insulator as a CMOS‐compatible physical reservoir [101]. Owing to the dynamic characteristics of ferroelectric polarization and polarization‐charge interaction, the FeFET can convert time‐series input into current values to form the reservoir state. After training, the system successfully realized the nonlinear time series prediction task with low cost. To extract features more effectively and further enhance RC's computing performance, Liu et al. constructed a multiscale RC system using the *α*‐In_2_Se_3_‐based FeFET [47]. The relaxation time of the ferroelectric device can be tuned easily with the back‐gate voltage, which can significantly enhance the richness of the reservoir state and further extract the features of different scales (Figure 3f). Ultimately, the system successfully achieved the processing of multiple superimposed oscillator tasks and significantly improved the robustness of time‐series prediction. Furthermore, a multilayer RC system was successfully implemented by connecting the *α*‐In_2_Se_3_ based FeFET to a planar *α*‐In_2_Se_3_ device, as shown in Figure 3g [102]. In this system, due to the resistance matching and voltage division between the two devices, both the input and output of the RC system are voltages, which thus allows cascading by connecting the output terminal of the first RC to the input terminal of the second. After training, this system can correctly classify sine and square waves with a normalized root mean square error of 0.2, demonstrating its superior high‐dimensional mapping capability and computational capability.

### 2.4 Simulation of Adaptive Long‐Term Plasticity and Its Application in Spatiotemporal Information Processing

The spatio‐temporal processing ability integrates different modal information through the coexistence of LTSP and STSP and historical adaptability. In a ferroelectric synaptic device, the integration of the volatile physical mechanisms and non‐volatile ferroelectric polarization switching processes enables the coexistence and dynamic regulation of LTSP and STSP. By integrating the history adaptation characteristics into long‐term synaptic behavior to form the adaptive long‐term synaptic plasticity, such devices can not only successfully recapitulate classical neural learning algorithms but also effectively simulate the biological nervous system's ability to encode and process spatio‐temporal correlation information [103, 104].

**FIGURE 4:**
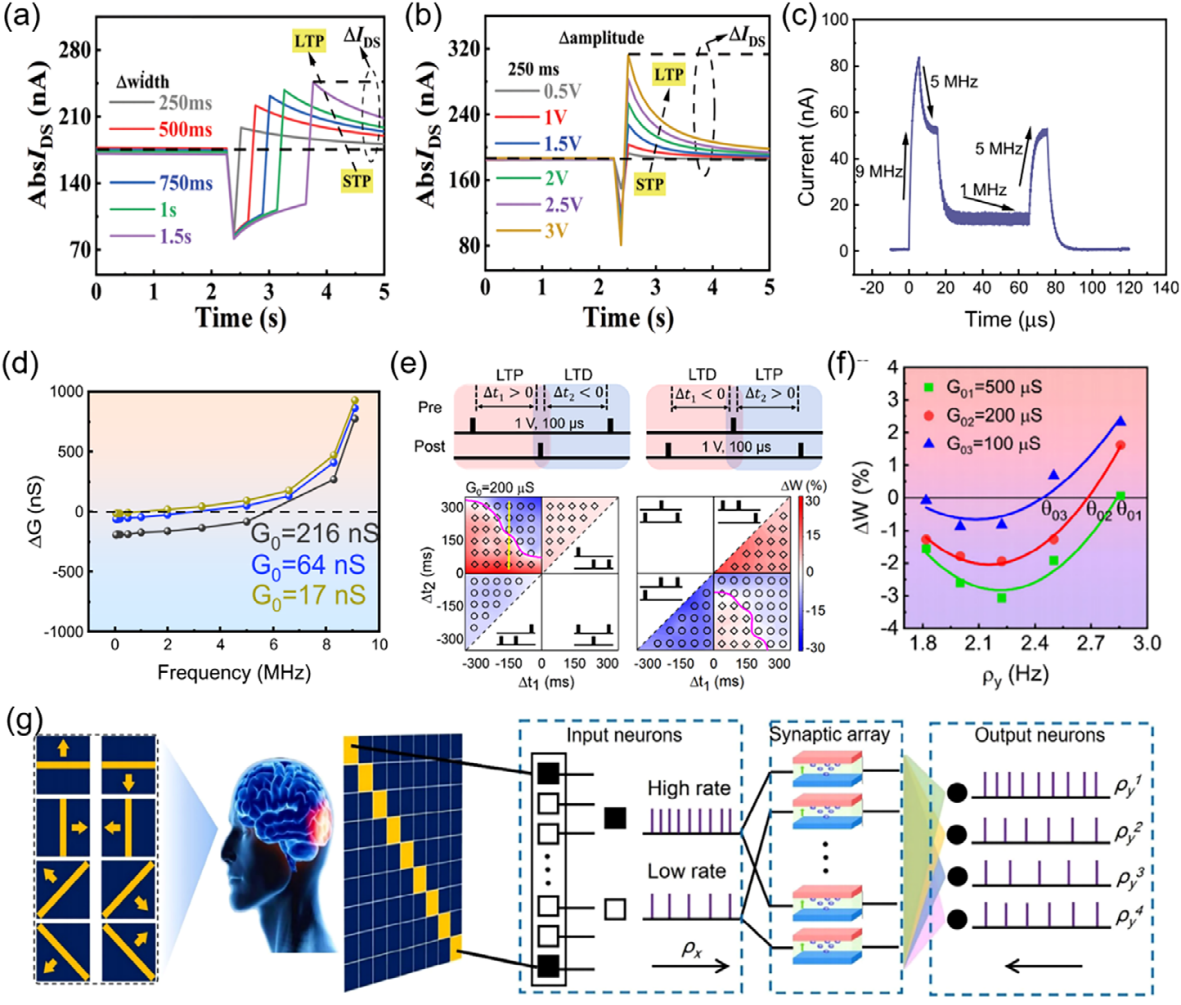
The adaptive long‐term synaptic plasticity in ferroelectric devices and its application in spatiotemporal information processing. (a,b) The dependence of EPSC on historical pulse with and amplitude. Reproduced with permission [105]. Copyright 2023 John Wiley and Sons. (c) The adaptation behavior and (d) the sliding threshold frequency effect of history‐dependent synaptic adaptation. Reproduced with permission [106]. Copyright 2024 American Chemical Society. (e) The experimental results of triplet‐STDP with initial state *G*_0_ = 200 µS (bottom panel). Reproduced with permission [100]. Copyright 2025 John Wiley and Sons. (f) BCM learning rule based on triplet‐STDP with different initial state. Reproduced with permission [100]. Copyright 2025 John Wiley and Sons. (g) Schematic diagram of the feedforward neural network designed for direction and moving direction recognition. Reproduced with permission [100]. Copyright 2025 John Wiley and Sons.

A typical illustration is presented in Figure 4a,b, by varying the pulse parameters, the transition from STSP to LTSP was achieved in the P(VDF‐TrFE‐CFE) based FET [105]. It was found that the final non‐volatile conductance states of the devices depend strongly on the parameters such as the width and amplitude of historically experienced pulses. Notably, the frequency difference of historical stimulations would also affect the subsequent synaptic plasticity in the ferroelectric devices. As exhibited in Figure 4c, it is evident that at 5 MHz (second phase and fourth phase) the synaptic plasticity was completely different [106]. The former and latter 5 MHz spike train induced “synaptic depression” and “synaptic potentiation”, respectively, depending on the different historical stimulations (9 and 1 MHz), which further confirms that the adaptation behavior can be imitated in the Au/Cr/La_0.1_Bi_0.9_FeO_3_/La_0.7_Sr_0.3_MnO_3_/SrTiO_3_ FTJs. Figure 4d further depicts the dependence of synaptic weight △*G* on frequency for different initial conductance *G*_0_. Generally, low‐frequency pulses trigger synaptic depression, while high‐frequency stimuli result in synaptic potentiation. As *G*_0_ increases from 17 to 216 nS, the frequency threshold (the frequency where △*G* = 0 happens) shifts significantly from 0.8 to 5.6 MHz, corresponding to the sliding threshold frequency effect of history‐dependent synaptic adaptation.

The successful implementation of adaptive long‐term synaptic plasticity and sliding threshold frequency implies that the Bienenstock‐Cooper‐Munro (BCM) rule, a learning rule for spatiotemporal processing can be effectively mimicked in the ferroelectric devices [107, 108]. This achievement paves the way for the high‐order spatiotemporal coding and learning of ferroelectric memristive networks. First, the triplet‐STDP, as the foundation for generating BCM learning rules, has recently been successfully replicated in the Au/Cr/BaTiO_3_/Nb:SrTiO_3_ FTJ (Figure 4e) [100]. Furthermore, different BCM curves can be obtained by extracting the data in Figure 4e and changing the initial state of the device (Figure 4f). By using the learning rules of BCM to realize the direction selection function, the neural network based on the FTJs successfully realized the direction selection and movement direction recognition functions of the visual cortex (Figure 4g). The pioneering work by Nie et al. further validates the potential of ferroelectric devices in processing spatio‐temporal recognition tasks by closely replicating the computational functions of the human brain.

## 3 Ferroelectric Devices for In‐Sensor Computing

In‐sensor computing is an emerging paradigm that integrates sensing, memory, and computation within a single device, allowing information acquisition and processing to be performed directly at the sensor level [109, 110]. By minimizing data movement across the interface between the sensor and the processor, this architecture significantly reduces system power consumption and latency while improving compactness and efficiency. From a sensor‐centric computing perspective, current approaches can be broadly classified into near‐sensor and in‐sensor computing [5]. Near‐sensor computing reduces data‐transfer overhead by collocating processing units with sensors, while in‐sensor computing eliminates the boundary between sensing and processing units by embedding memory and computation directly within the sensing device [42, 111, 112, 113, 114], enabling energy‐efficient preprocessing directly during signal transduction. Among various devices explored for emerging neuromorphic computing, memristors [115, 116], RRAM, PCM, MRAM, and photosensitive transistors have all demonstrated potential for sensing and computing applications. In contrast to the charge trapping/ion migration, resistive switching, amorphous‐to‐crystalline transition, magnetization switching, and light‐gating effects used in these devices, ferroelectric devices rely on intrinsic polarization switching to achieve non‐volatile and continuously tunable conductance states. Ferroelectric devices can convert optical, thermal, and mechanical signals into their own polarization states through the photovoltaic, pyroelectric, and piezoelectric effects, enabling them to directly sense physical stimuli. This direct coupling stems from the intrinsic properties of ferroelectrics, distinguishing them from other platforms that depend on defect engineering or structural design. In addition, the multi‐field coupling capability further allows ferroelectric devices to integrate multiple sensory modalities within a single device, offering a compact platform for multimodal in‐sensor computing. The programmable multi‐state nature of polarization thereby provides stable internal electrical states that inherently support simultaneous sensing, memory, and local computation. Depending on the sensing modality, this chapter is organized into three sections, visual, tactile, and multimodal sensing systems, and it systematically reviews the key research progress of ferroelectric devices in these fields.

### 3.1 Visual Sensing Systems

Within in‐sensor computing systems, the perception and preprocessing of optical signals are crucial for real‐time interpretation of complex scenes, supporting applications such as autonomous driving and intelligent surveillance [117]. Ferroelectric devices convert optical signals into electrical responses that reconfigure ferroelectric polarization, and the resulting remanent polarization non‐volatilely encodes the information, providing a stable basis for local computation and sensing [118, 119]. Depending on how visual information is processed, in‐sensor computing systems can be broadly classified into static and dynamic vision systems. Static systems focus on spatial feature extraction and recognition from single‐frame images, while dynamic systems process temporal information and motion across consecutive frames. The principles and representative advances of these two categories are detailed below.

#### 3.1.1 Static Vision Systems: Image Recognition and Classification

Static vision systems provide a fundamental basis for spatial feature extraction in machine vision and support tasks such as object recognition and image classification. Based on device structure, ferroelectric sensing devices can be broadly classified into two‐terminal and three‐terminal architectures.

**FIGURE 5:**
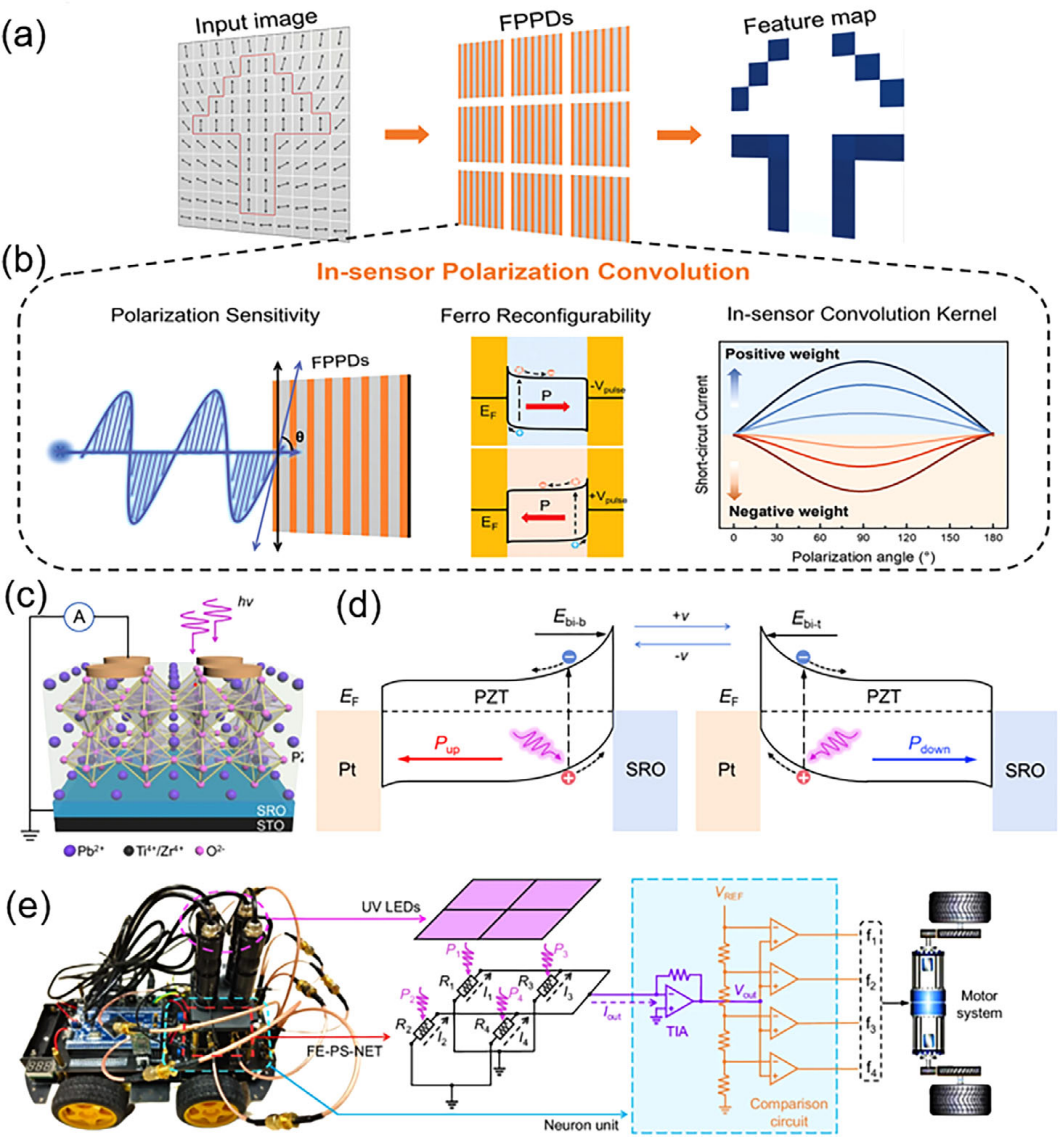
Two‐terminal ferroelectric sensing devices for static vision systems. (a) Polarization‐encoded feature map extraction using FPPDs, where arrow directions denote incident light polarization. Reproduced with permission [120]. Copyright 2025 John Wiley and Sons. (b) Polarization‐sensitive photoresponse enabled by aligned BFO nanowire arrays (left), ferroelectric‐polarization‐tunable photovoltaic band modulation (middle), and non‐volatile photocurrent weights for in‐sensor convolution enabled by the reconfigurable photovoltaic effect (right). Reproduced with permission [120]. Copyright 2025 John Wiley and Sons. (c) Schematic of the device structure of the Pt/PZT/SRO FE‐PS. Reproduced with permission [121]. Copyright 2025 Spring Nature. (d) Illustration of the polarization‐controlled photoresponse via Schottky barrier modulation. Reproduced with permission [121]. Copyright 2025 Spring Nature. (e) Photograph of the vehicle and schematic circuit diagrams showing the FE‐PS‐NET. Reproduced with permission [121]. Copyright 2025 Spring Nature.

For two‐terminal ferroelectric devices, as shown in Figure 5a, Wang et al. developed a ferroelectric‐reconfigurable polarization‐sensitive photodiode (FPPD) based on BiFeO_3_ nanowire arrays, establishing a foundation for in‐sensor computing along the polarization dimension [120]. The device senses the polarization angle of incident light by exploiting the structural anisotropy of the nanowires, which creates a polarization‐dependent carrier profile. This profile is then converted into a programmable photocurrent governed by the ferroelectric photovoltaic effect via tunable Schottky barriers (Figure 5b). This polarization‐sensitive detection enhances recognition reliability under adverse conditions.

Beyond encoding optical attributes such as polarization, static visual perception further requires array‐level representation of spatial information. Building on these advances, Lin et al. constructed an in‐sensor platform based on ferroelectric photosensors (FE‐PSs), enabling high‐speed recognition of traffic sign images for autonomous driving [121]. As shown in Figure 5c, the system is built on Pt/Pb(Zr_0_._2_Ti_0_._8_)O_3_/SrRuO_3_ (Pt/PZT/SRO) unit cells, each operating in a self‐powered photovoltaic mode to generate photocurrents. Critically, the magnitude and sign of these photocurrents are directly controlled by the nonvolatile remanent polarization state, enabling a self‐powered, symmetric, and non‐volatile photoresponse (Figure 5d). To program this response with high precision, a bidirectional closed‐loop scheme applies and verifies voltage pulses, compensating for device nonlinearity to achieve accurate photocurrent tuning. Integrated into a vehicle (Figure 5e), the FE‐PS‐NET feeds these photocurrents to a neuron module and then to the motor system, enabling real‐time traffic sign recognition.

**FIGURE 6:**
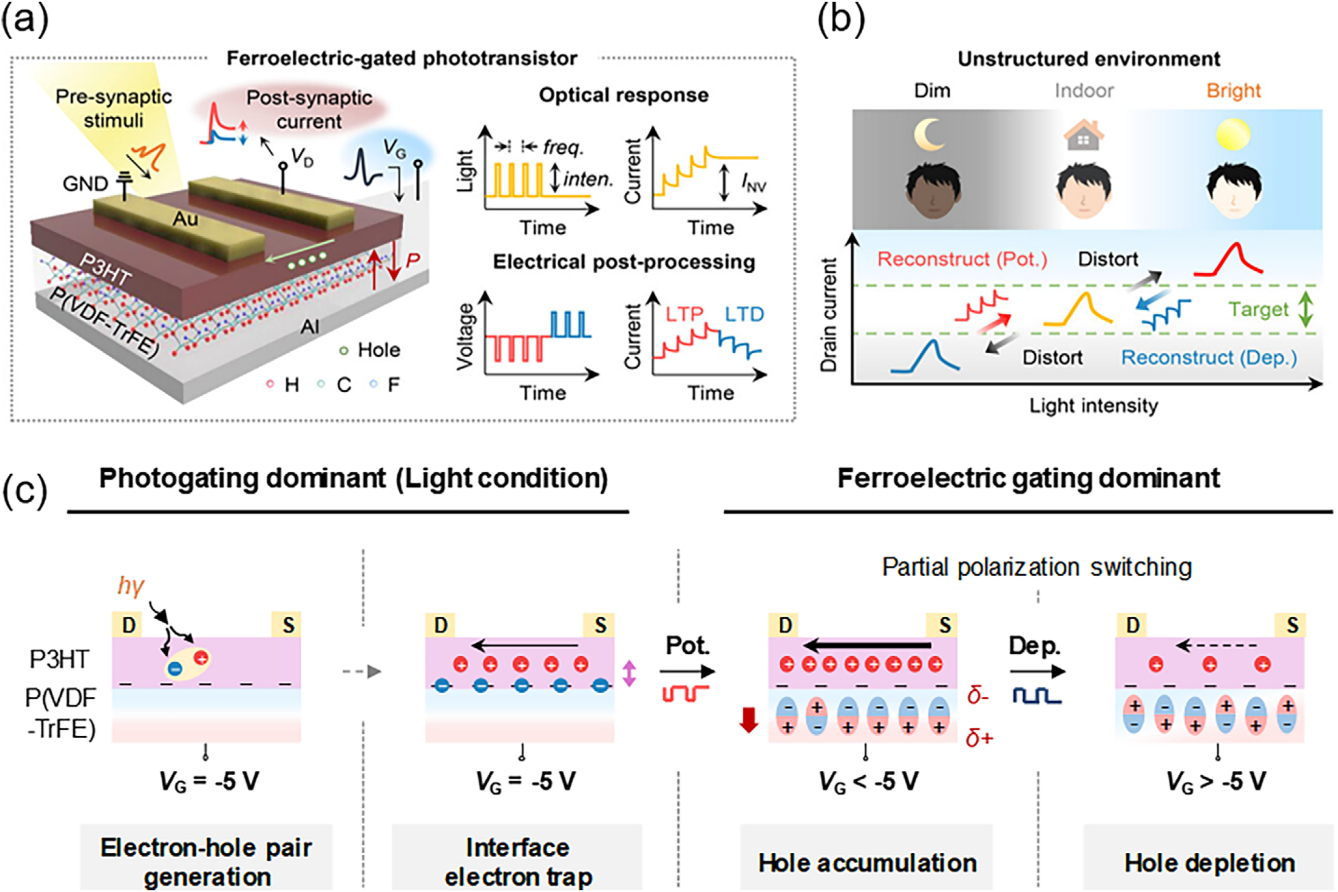
Three‐terminal ferroelectric sensing devices for static vision systems. Reproduced with permission [122]. Copyright 2025 John Wiley and Sons. (a) Schematic of the FGPT with P3HT photoactive channel and a P(VDF‐TrFE) ferroelectric gate insulator. (b) Conceptual diagram of the in‐sensor processing system for all‐day face recognition under varying illumination. (c) Schematic illustration of electron and hole dynamics under photogating‐dominant and ferroelectric‐gating‐dominant conditions.

To further enhance adaptive visual perception, as illustrated in Figure 6a, Lee et al. developed a three‐terminal ferroelectric‐gated phototransistor (FGPT) capable of in‐sensor adaptive calibration of light intensity signals, enhancing face recognition robustness under extreme illumination (Figure 6b) [122]. The device employs a bottom‐gate structure with a ferroelectric polymer P(VDF‐TrFE) as the gate dielectric and an organic semiconductor poly(3‐hexylthiophene) (P3HT) as the photoactive channel. The photogating effect, through charge trapping at the ferroelectric–semiconductor interface, directly converts light intensity into persistent non‐volatile conductance for storage, effectively encoding optical input at the device level. Through gate‐voltage‐controlled partial polarization switching of ferroelectric domains, the stored conductance can be linearly tuned in real time (Figure 6c). When extreme illumination causes the device current to deviate from a set threshold, applied gate voltage pulses realign the photoconductance with the adaptable range of a pre‐trained model. This enables the device to adapt in real time to changes in light intensity without retraining. This mechanism improves face recognition accuracy by approximately 40% without algorithmic retraining and highlights the hardware advantage of ferroelectric devices for adaptive intelligent perception.

Based on the above representative works, we compare the two‐terminal and three‐terminal architectures as follows: In two‐terminal devices, electrical modulation (i.e., applying voltage pulses to adjust the light response sensitivity) and light sensing share the same pair of electrodes, so they cannot operate simultaneously. Since the device cannot be dynamically adjusted by electric pulses during light sensing, such devices are more suitable for static scenarios. The advantages of these devices lie in their simple structure and high‐density integration. Additionally, some two‐terminal devices (e.g., FE‐PS) achieve low energy consumption due to their self‐powered operation [121].

In three‐terminal devices, the independent gate separates the electrical modulation and light sensing paths, which increases structural complexity and may result in slightly lower integration density. However, this separation lays the groundwork for the potential simultaneous operation of the two functions in the future. Therefore, three‐terminal devices are better suited for dynamic environments that require real‐time adaptation to changing light conditions.

#### 3.1.2 Dynamic Vision Systems: Motion Detection and Temporal Processing

Dynamic vision systems are essential for enabling intelligent visual perception. Their primary function is to extract spatiotemporal features from continuous image sequences, supporting tasks such as motion detection and trajectory tracking [123]. Existing dynamic vision systems can be broadly classified into two categories based on their functional focus. The first emphasizes motion pattern recognition by interpreting the temporal evolution of signals, while the second identifies and segments moving objects by analyzing pixel‐level differences between consecutive frames [124, 125].

**FIGURE 7:**
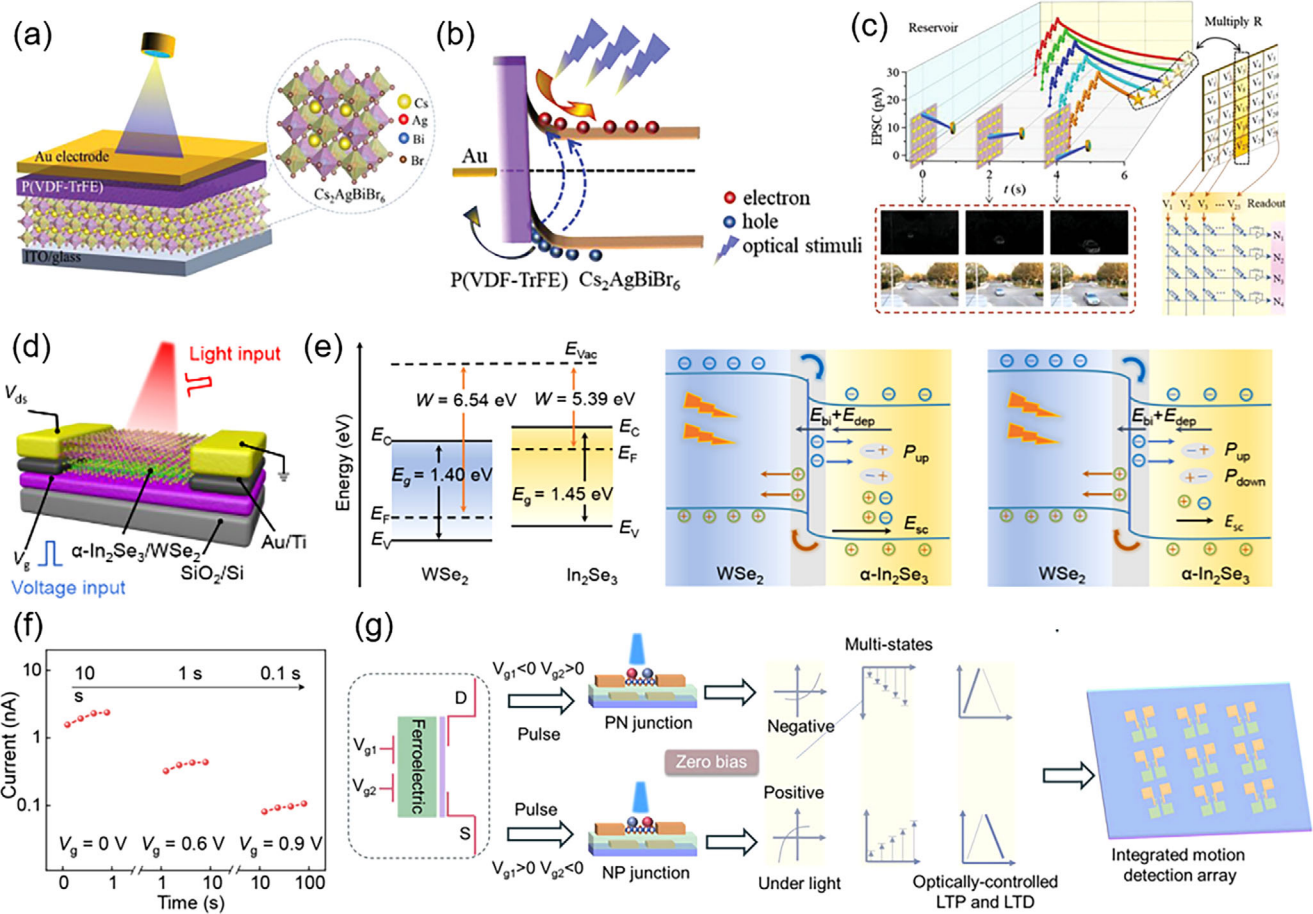
Ferroelectric sensing devices for dynamic vision systems. (a) Schematic of the photonic device and Cs_2_AgBiBr_6_ crystal structure (inset). Reproduced with permission [126]. Copyright 2022 John Wiley and Sons. (b) The band diagram of the device under optical stimulus. Reproduced with permission [126]. Copyright 2022 John Wiley and Sons. (c) A concrete example of the reservoir array responding to the up‐to‐down vehicle flow. Reproduced with permission [126]. Copyright 2022 John Wiley and Sons. (d) The diagram of the α‐In_2_Se_3_/WSe_2_ heterojunction devices. Reproduced with permission [127]. Copyright 2025 American Chemical Society. (e) Schematics showing the type‐II band alignment in the α‐In_2_Se_3_/WSe_2_ heterostructure, the built‐in‐field‐driven separation of photogenerated carriers, and the resulting screening‐induced weakening of the internal electric field. Reproduced with permission [127]. Copyright 2025 American Chemical Society. (f) Gate‐tunable temporal processing of four optical pulses by the FePNJ. Reproduced with permission [127]. Copyright 2025 American Chemical Society. (g) Vision sensor with PN and NP configurations, enabling nonvolatile intermediate negative and positive photocurrents. Reproduced with permission [128]. Copyright 2024 American Chemical Society.

As a representative of the first category, Lao et al. developed a self‐powered Au/P(VDF‐TrFE)/Cs_2_AgBiBr_6_/ITO sensor array for reservoir computing to recognize dynamic vehicle flow directions (Figure 7a) [126]. The perception layer consists of a 5×5 sensor array. The system's key nonlinear spatiotemporal mapping capability arises from the trapping of photogenerated carriers at the potential wells of the ferroelectric polymer interface (Figure 7b). The sensor array converts moving light signals into changing currents that reflect the direction of vehicle flow, allowing the system to detect motion directly at the device level (Figure 7c).

Real‐world motion occurs at varying speeds, requiring sensing systems with adaptive temporal processing ability. To address this challenge, Li et al. developed a ferroelectric *p–n* junction (FePNJ) by forming a heterojunction between the in‐plane ferroelectric semiconductor α‐In_2_Se_3_ and WSe_2_, integrating light sensing, dynamic signal processing, and information storage in a single device (Figure 7d) [127]. The interplay between ferroelectric polarization and the built‐in electric field governs photogenerated carrier separation and recombination: incident light generates electron–hole pairs in the heterojunction, and the internal electric field, modulated by ferroelectric polarization, drives their spatial separation. This mechanism converts optical motion signals into electrical responses whose amplitude and decay dynamics can be tuned, enabling control over temporal processing across a wide range (Figure 7e). Gate‐voltage control enables relaxation times to be adjusted over a wide range from 0.1 to 1000 s (Figure 7f). Negative gate voltage provides a long relaxation time that retains previous frame signals over an extended period, enabling the device to preserve temporal information for slow motion perception such as a person walking. In contrast, positive gate voltage delivers a short relaxation time that refreshes signals rapidly, allowing the device to capture fast‐changing scenes and detect fast‐moving objects such as a car. This tunable characteristic enables the system to encode motions at different time scales, making it suitable for real‐world applications such as real‐time traffic monitoring. By overcoming the fixed temporal resolution limitation of conventional sensors, the system achieves high‐quality spatiotemporal information encoding for multiscale dynamic vision tasks.

The inter‐frame difference method represents another important paradigm for motion processing. Dang et al. employed a three‐terminal WSe_2_/P(VDF‐TrFE) optoelectronic sensor to detect and recognize moving targets by sensing differences between consecutive image frames [128]. The device uses two independent gates to locally induce *p*‐type or *n*‐type accumulation regions in the WSe_2_ channel via ferroelectric polarization, forming reconfigurable *p–n* or *n–p* homojunctions. Under illumination, these junctions generate self‐powered, bidirectional photocurrents with highly linear and symmetric multilevel states (Figure 7g). By performing analog‐domain computation between consecutive frame signals and these positive–negative photocurrent matrices, the system suppresses background signals while highlighting moving objects, achieving 96.8% detection accuracy. Following a similar technical paradigm, Chen et al. extended this approach using an InSe/ferroelectric‐PZT heterostructure to achieve broadband optical sensing across 450–973 nm, substantially expanding the operational spectrum for dynamic visual detection sensors [129].

### 3.2 Tactile Sensing Systems

Tactile signals are a crucial source of information for human‐machine interaction and intelligent electronic skins (E‐skins), demonstrating unique value in the field of in‐sensor computing [130, 131]. The primary goal of tactile in‐sensor computing is to implement a complete functional chain from pressure perception to information memory within a single device. Pressure or touch stimuli directly encode tactile information by modulating the non‐volatile polarization of ferroelectric materials. This encoded polarization state produces persistent changes in the device's electrical output, thus enabling bio‐inspired tactile systems with adaptive learning capabilities.

**FIGURE 8:**
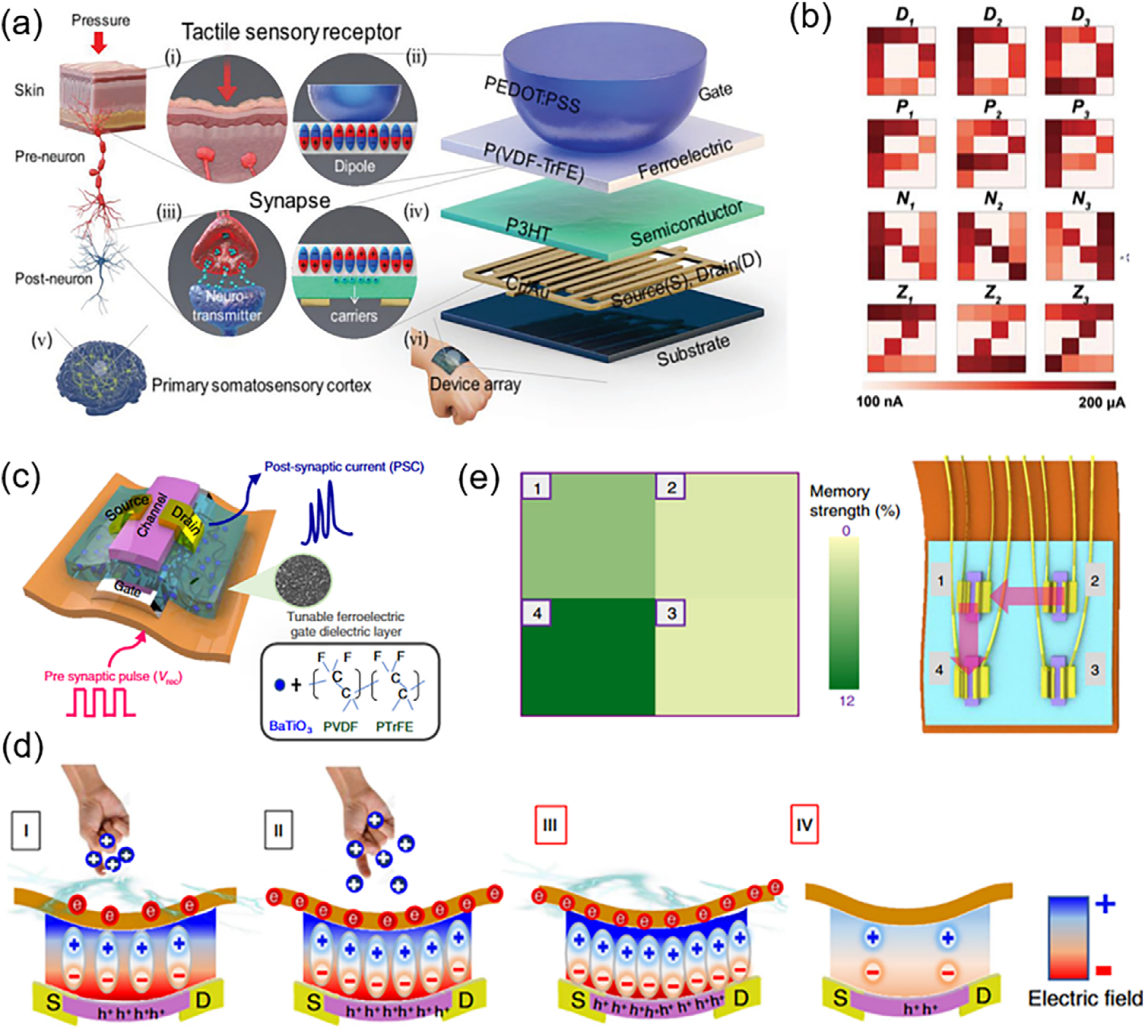
Tactile sensing systems. (a) Bio‐inspired tactile perception scheme (left) and the corresponding FeFET‐based artificial tactile e‐skin (right). Reproduced with permission [132]. Copyright 2020 John Wiley and Sons. (b) PSC contour maps for four letters across three handwriting styles. Reproduced with permission [132]. Copyright 2020 John Wiley and Sons. (c) Schematic of the flexible OFST gated by a triboelectric‐capacitive coupling effect. Reproduced with permission [130]. Copyright 2020 Spring Nature (CC BY). (d) Operating principles of the transistors for pressure stimuli. Reproduced with permission [130]. Copyright 2020 Spring Nature (CC BY). (e) Schematic of pixel memory magnitude mapping in a 2 × 2 array. Reproduced with permission [130]. Copyright 2020 Spring Nature (CC BY).

Based on the pressure‐modulated ferroelectric polarization mechanism, Lee et al. developed a dome‐gate FeFET tactile sensor capable of detecting both pressure magnitude and pulse number, enabling recognition of handwritten letters (Figure 8a) [132]. The device employs a P(VDF‐TrFE) ferroelectric polymer as the gate dielectric. Its working principle relies on deformation of the dome‐shaped gate to modulate the effective contact area with the ferroelectric layer; combined with an applied gate voltage, this controls the degree of ferroelectric domain switching. Pressure inputs are thus directly mapped onto stable, non‐volatile changes in channel conductance. A 4 × 4 array based on this device was used to encode handwritten letters, achieving a recognition accuracy of 99.66% after 10 training epochs, even under 10% noise interference (Figure 8b).

Building upon the triboelectric‑ferroelectric coupling mechanism, a flexible organic ferroelectric synaptic transistor (OFST) has been developed as an integrated tactile sensing unit, capable of perceiving and memorizing spatiotemporal touch sequences (Figure 8c) [130]. The device employs a barium titanate nanoparticle (BT NPs)/P(VDF‐TrFE) ferroelectric nanocomposite as the gate dielectric. The triboelectric potential generated upon finger touch is harnessed to drive polarization switching in the ferroelectric layer (Figure 8d), thereby transducing pressure stimuli into non‐volatile channel conductance changes. In a 2 × 2 array, these devices can record touch history and infer the order of touches by analyzing post‐stimulus conductance, which reflects the relaxed polarization state (Figure 8e). These works illustrate the capability of ferroelectric devices to integrate tactile sensing and memory at the device level, enabling compact, information‐rich tactile systems.

### 3.3 Multimodal Sensing System

In complex environments, single‐modality perception often faces limitations. Ferroelectric‐based multimodal in‐sensor computing overcomes these challenges by allowing a single device to simultaneously sense and process multiple physical signals such as light, pressure, and temperature, enabling more comprehensive and robust perception [133, 134].

**FIGURE 9:**
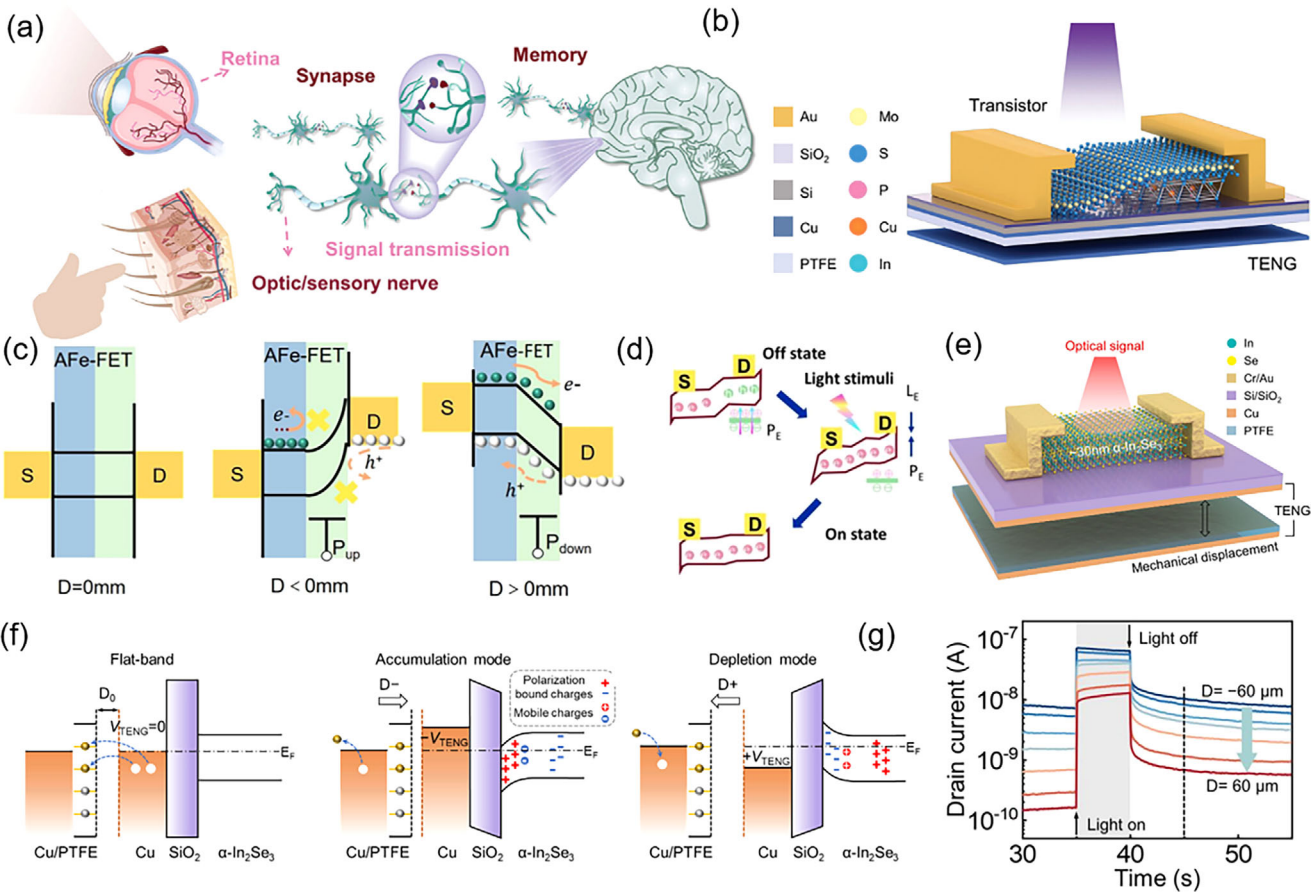
Multimodal sensing systems. (a) Schematic diagram of the human tactile/visual sensory system. Reproduced with permission [135]. Copyright 2024 John Wiley and Sons. (b) Diagram of the asymmetric MoS_2_ /CIPS heterostructure. Reproduced with permission [135]. Copyright 2024 John Wiley and Sons. (c) Corresponding energy band diagram of the tribotronic transistor in different states. Reproduced with permission [135]. Copyright 2024 John Wiley and Sons. (d) Operation mechanism of device for photoelectrical memory. Reproduced with permission [135]. Copyright 2024 John Wiley and Sons. (e) Schematic of the tribo‐ferro‐optoelectronic neuromorphic device. Reproduced with permission [138]. Copyright 2023 John Wiley and Sons (CC BY). (f) Energy band diagram of the tribotronic α‐In_2_Se_3_ transistor in different states. Reproduced with permission [138]. Copyright 2023 John Wiley and Sons (CC BY). (g) Current responses of α‐In_2_Se_3_ to light pulses in different ferroelectric polarization states. Reproduced with permission [138]. Copyright 2023 John Wiley and Sons (CC BY).

Drawing inspiration from the biological multisensory integration shown in Figure 9a, Gong et al. developed a multimodal sensing device based on an asymmetric MoS_2_/CIPS ferroelectric heterostructure (Figure 9b) [135]. In this device, the ferroelectric polarization state of the CIPS layer serves as the primary non‐volatile information‐storage and processing medium. Mechanical displacement (Figure 9c) is converted into a gate voltage through the integrated triboelectric nanogenerator (TENG), while light produces photocarriers via the photovoltaic effect, both of which jointly program the CIPS polarization. This enables simultaneous fusion and in situ processing of the two distinct physical signals within a single ferroelectric medium (Figure 9d). Under combined stimulation, the device exhibits nonlinear synergistic enhancement and can implement basic logic functions, such as AND operations. These results demonstrate that ferroelectric materials can directly transduce multiple external signals into programmable polarization states for in‐sensor computation. Based on the same CIPS ferroelectric platform, Zhang et al. further constructed a ReS_2_/CIPS heterojunction transistor [136]. They simulated the tactile and visual synaptic behaviors through coordinated weight updates and achieved an accuracy of 95.46% in handwritten digit recognition based on CNN.

Building on this work, the same team replaced CIPS with *α*‐In_2_Se_3_ and fabricated a WSe_2_/*α*‐In_2_Se_3_ heterojunction [137]. Mechanical displacement dynamically switches the ferroelectric polarization of *α*‐In_2_Se_3_, enabling non‐volatile reversible switching between *p*‐*n* and n‐p junctions. In the *p*‐*n* state, the built‐in field generates a strong photocurrent. In the *n*‐*p* state, the reversed field suppresses it. Thus, the same optical input yields drastically different outputs depending on the mechanical history, demonstrating displacement‐dependent photoresponse modulation. Based on this mechanism, the device achieves self‐powered photodetection at zero bias and Boolean logic operations with mechanical displacement and light as dual inputs.

In contrast to heterojunction‐based integration, Feng et al. demonstrated a multimodal sensing device using the ferroelectric semiconductor *α*‐In_2_Se_3_ (Figure 9e), capable of directly detecting both optical and mechanical displacement signals [138]. For optical inputs, *α*‐In_2_Se_3_ serves as a photosensitive channel that generates charge carriers, while an integrated triboelectric unit converts mechanical displacement into a gate potential that switches the ferroelectric polarization (Figure 9f). This monolithic design allows photogenerated carriers and ferroelectric polarization changes to interact within a single material. Optical signals modulate the channel charge density, while mechanical signals reconfigure the internal electric field via polarization control; together, these effects produce electrical responses that reflect the combined stimuli (Figure 9g). Furthermore, the device can associate the two sensory modalities, demonstrating higher‐level multisensory information processing and moving beyond basic in‐sensor signal integration.

These four works explore multimodal perception from different perspectives. Gong and Zhang et al. demonstrated synergistic modulation of mechanical and optical signals [135, 136]. Zhang et al. further demonstrated a displacement‐dependent photoresponse modulation [137]. Feng et al. utilized a single material to integrate multimodal responses into one system [138]. Collectively, these studies illustrate an evolution from synergistic modulation to mechanically dominated modulation and finally to integration. They provide concrete examples for understanding multimodal signal interaction at the device level and offer diverse strategies for future multimodal perception systems. Recent work has further demonstrated multisensory information processing at the system level, achieving cross‐modal recognition and non‐empirical cognitive functions [25]. This offers a reference for future system‐level integration of the ferroelectric devices discussed above.

## 4 Challenges and Prospects

**FIGURE 10:**
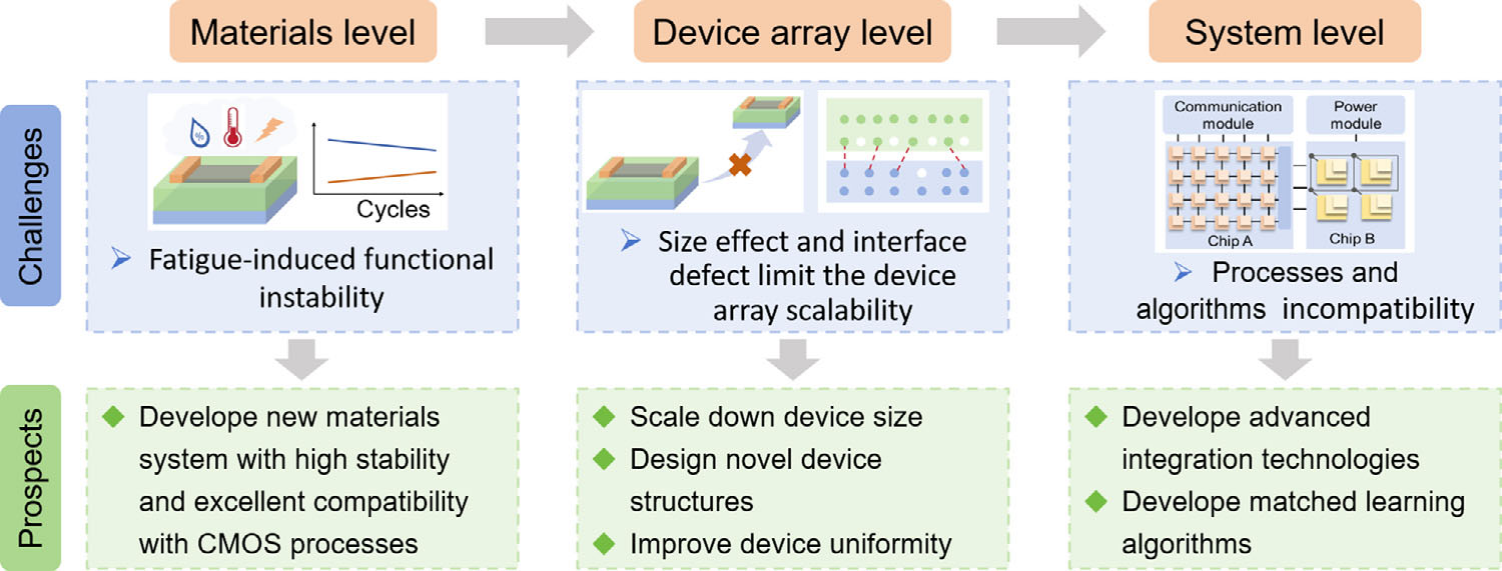
Challenges and prospects of the ferroelectric neuromorphic devices for future practical application.

In summary, ferroelectric neuromorphic devices provide a robust hardware foundation for the construction of efficient neuromorphic computing architectures. This paper mainly reviews the basic structure and working mechanisms of the ferroelectric neuromorphic devices and their cutting‐edge research advances in in‐memory computing and in‐sensor computing architectures. While existing researches have fully demonstrated the great application potential of ferroelectric devices in the aforementioned fields, most remain at the proof‐of‐concept stage. In this section, we will systematically discuss the key obstacles that hindering the practical application of ferroelectric neuromorphic devices and propose optimization strategies from the perspectives of materials, device arrays and system integration (Figure 10).

### 4.1 Challenges

In future large‐scale practical application and industrialization, several challenges and limitations across multiple level will continue to restrict the evolution of ferroelectric neuromorphic devices, including functional stability, device array scalability, and system compatibility, thereby limiting their ultimate performance, energy efficiency, and reliability.

1. **Functional stability**. For practical application, the devices must maintain their intended performance over time and under varying environmental conditions. However, the fatigue of ferroelectric materials is the key factor compromising the device stability. During cyclic operation, the migration and clustering of charged defects would pin domain walls and hinder polarization switching, ultimately leading to device failure [139, 140]. Furthermore, environmental factors can exacerbate this instability. For instance, in high‐density integrated circuits, the concentration of current, heat, and stress will accelerate the ion migration, defect generation, significantly degrading polarization endurance. Moreover, the adsorption of moisture from the environment onto the ferroelectric surface may introduce unintended built‐in electric fields, compromising polarization retention, thereby undermining the long‐term reliability.
2. **Device array scalability**. Practical implementation requires the fabrication of large‐scale and high‐density device arrays. However, the size effect of ferroelectric materials is the core constraint of device miniaturization and high‐density integration. When the conventional ferroelectric films are thinned down to a few nanometers in thickness or scaled below tens of nanometers laterally, ferroelectric properties degrade significantly and may even vanish below a critical size. Moreover, during miniaturization, non‐ideal factors such as defects and stress at the electrode/ferroelectric interface become more pronounced, increasing performance variations and noise among devices. This affects the uniformity of the array and ultimately limits the controllable fabrication of large‐area high‐density arrays.
3. **System compatibility**. For system‐level applications, ferroelectric neuromorphic devices must be heterogeneously integrated with other functional components, interfaced with peripheral circuitry, and remain compatibility with machine learning algorithms. First, process compatibility issues arise when integrating with other functional devices. Second, the conventional perovskite ferroelectric materials typically require high‐temperature during fabrication, which conflicts with the low‐temperature constraints of CMOS processes. Furthermore, widely used machine learning algorithms (e.g., backpropagation), which are designed under idealized hardware assumptions, cannot adequately adapt to or tolerate the inherent non ideal characteristics of ferroelectric devices, such as device‐to‐device variability and operational noise.

### 4.2 Prospects

Challenges and constraints often give rise to new strategies and opportunities. Currently, the key to addressing the issues mentioned above lies in collaborative optimization across multiple levels, from materials and device arrays to system integration.

**At the material level**, it is essential to develop new ferroelectric materials system with high stability and good compatibility with CMOS processes. For instance, the sliding ferroelectric materials will provide a novel pathway to address the long‐standing issue of fatigue in ferroelectrics [141]. Additionally, hafnium‐based ferroelectrics and two‐dimensional ferroelectric materials have attracted considerable attention due to their intrinsic CMOS compatibility and excellent dimensional scalability [142, 143]. Similarly, the AlScN system with sustainable scaling characteristics, which also offers new selection for performance optimization [144]. However, the internal physical mechanism of these new ferroelectric materials (such as domain reversal dynamics) and the preparation processes for high‐quality, large‐area still require systematic exploration and optimization.

**At the device array level**, emphasis should be placed on scaling down device size and improving uniformity. (1) For miniaturization, a fundamental strategy involves nanoscale grain engineering within the ferroelectric domain while maintaining the ferroelectric properties, which requires in‐depth investigation of crystallization dynamics and optimized processing. Concurrently, the exploration of novel device architectures utilizing the emerging ferroelectric material systems provides complementary pathways for size reduction. (2) In addition, to enhance device uniformity, improving interface quality is critical for suppressing defect formation and minimizing performance variations. This is accomplished through precise control of crystallization kinetics, deliberate interface engineering, and the implementation of rapid, uniform annealing processes. Thus, more research is required at the device array level to realize miniaturization, high density, and excellent uniformity in ferroelectric devices. It is worth emphasizing that continuous optimization at this level not only directly enhances device array performance but also extends its impact to the algorithmic level [26]. It is anticipated that the optimized devices will provide more efficient hardware support for machine learning algorithms, thereby laying a more solid computational foundation for handling complex tasks.

**At the system level**, the focus should shift to developing advanced integration technologies and matched learning algorithms. Specifically, this involves: (1) Exploring novel integration schemes to overcome the heterogeneous interconnection bottlenecks among different functional components, thereby enabling efficient connectivity in large‐scale arrays of artificial neurons and synapses. (2) Designing neuron and synapse circuit modules that align with ferroelectric device characteristics (e.g., nonlinear conductance and dynamic response) and support complex learning rules as well as the dynamic properties of neural networks. (3) Developing robust learning algorithms capable of adapting to non‐ideal device behaviors. It is noteworthy that technological advancements at this level exhibit synergistic development with machine learning algorithm development [26]. By leveraging machine learning methods to optimize device performance, design circuit model and refine integration processes, the progress of integrated systems can be significantly accelerated. Through the co‐design of algorithms and hardware, it is expected to enhance both system fault tolerance and learning efficiency, thereby achieving powerful on‐chip learning capabilities.

Notwithstanding the above technological challenges, ferroelectric neuromorphic devices, with their unique advantages and broad applicability, have demonstrated great promise in the field of bio‐inspired computing and have already emerged as the cornerstone of next‐generation electronic/optoelectronic neuromorphic systems. Looking ahead, continuous technological breakthroughs in this field are expected to overcome the inherent limitations of traditional silicon‐based technologies, thereby opening up new pathways for the sustainable evolution of modern electronic technologies.

## Funding

This work was supported by the National Natural Science Foundation of China (Grant/Award numbers: 12334006 and 12474088) and Natural Science Foundation of Shandong Province (Grant/Award numbers: ZR2022YQ43 and ZR2025QA07).

## Conflicts of Interest

The authors declare no conflict of interest.

## Data Availability

No new data were created or analyzed in this study.
